# Role of single or double ringed circumferential wound protectors in reducing surgical site infections following colorectal resections. A systematic review

**DOI:** 10.1016/j.amsu.2022.104656

**Published:** 2022-09-15

**Authors:** Hussameldin M. Nour, Amiya Ahsan, Dimitra V. Peristeri, Samuelson E. Osifo, Mr Krishna K Singh, Mr Muhammad S Sajid

**Affiliations:** a Department of Digestive Disease and General Surgery, Royal Sussex County Hospital, UHSussex NHS Trust, Eastern Road Brighton BN2 5BE, UK

**Keywords:** Wound protector, Colorectal surgery, Surgical wound infections, Surgical site infections

## Abstract

**Objective:**

The objective of this article is to explore whether the use of single or double ringed wound protectors (WP) in patients undergoing colorectal resection (CRR) are associated with reduced risk of surgical site infections (SSI).

**Materials and methods:**

Analysis was conducted according to PRISMA guidelines. With the help of expert local librarians, systematic search of medical databases like MEBASE, MEDLINE and PubMed was conducted to find appropriate randomized controlled trials (RCT) according to predefined inclusion criteria. The analysis of the pooled data was done using the principles of meta-analysis on statistical software RevMan version 5.

**Result:**

Twelve RCT on 2425 patients fulfilled the inclusion criteria. There were 1216 patients in the WP group and 1209 patients in the no-WP group. In the random effects model analysis, the use of WP during CRR was associated with the reduced risk of SSI [odds ratio 0.60, 95% CI (0.41–0.90), z = 2.49, P = 0.01]. However, there was significant heterogeneity (Tau^2^ = 0.22; Chi^2^ = 25.87, df = 11; (p = 0.007; I^2^ = 57%) among included studies.

**Conclusion:**

Use of WP seems to reduce the risk of SSI and therefore, may routinely be used during both open and laparoscopic CRR.

## Highlights

• Surgical site infections (SSIs) are a frequent complication following major abdominal surgery.
• The aim is to assess the effectiveness of using wound protectors in decreasing post operative SSIs in colorectal resection.
• Twelve RCTs were used to obtain the submitted data.
• Using wound protectors was associated with reduced post operative SSIs in colorectal resections.

## 1 Introduction

Surgical site infections (SSIs) are infections of the incision or organ or space that occur after surgery and they can be classified into superficial, deep or organ/space incisional infections [1]. SSIs are a frequent complication after major abdominal surgeries, affecting between 25% and 40% of patients [2] and it is also reported to be the most common hospital-acquired infection in Europe [3]. SSIs have a huge impact on surgical outcomes, they may lead to impaired wound healing, re-operation, increase use of antibiotics and increase hospital stay [4].This will also lead to further diagnostic tests and treatment, thus SSIs have a significant economic impact. It estimated that the financial impact in the UK is £30 million per year [5]. However, the mortality is low ranging between 1 and 4% [6].

As a result of the serious clinical and economic impact of SSIs, there are certain pre-operative and intraoperative measures which were developed to reduce post operative infections. Preoperatively, the use of prophylactic antibiotics was found to reduce the risk of post operative wound infection in colorectal surgery [7]. Also cleaning the hands and forearm prior to surgery helps to decrease the biocontamination of bacteria on the skin of the surgical team and stops the growth of bacteria [8]. In addition, the use of alcohol based preparation to sterilize the surgical field is helpful in reducing SSIs [8]. During the operation, wound irrigation [9], using antimicrobial impregnated sutures [10], wound protectors [11], maintaining normal blood pressure [11] and normal temperature [12] were found to be beneficial in reducing the risk of wound infection post operatively.

Wound protectors have been widely used in recent years, they are defined as plastic sheaths which cover the wound during surgery and help in retraction of an incision without the use of an extra mechanical retraction [13]. It has been suggested that the use of wound protectors helps to decrease the rate of SSIs in abdominal surgeries by protecting the wound edge from potential bacterial contamination [14].

The aim of the meta-analysis is to explore whether the use of single or double ringed wound protectors (WP) in patients undergoing colorectal resection (CRR) are associated with reduced risk of SSI.

## 2 Method

### 2.1 Data search

A thorough examination of various electronic archives such as MEDLINE, EMBASE and Cochrane Library was conducted in order to find appropriate trials which could be included in this meta-analysis. Furthermore, the titles which were obtained from the search were analysed to ensure their compatibility for potential inclusion or exclusion from the study. The references from chosen studies were also utilized as an extra source of searching to find RCTs. In this search, there was no restriction in using language, gender, sample size and origin of the study. To narrow and widen the results, Boolean operators (AND, OR, NOT) were liberally used.

### 2.2 Study selection

In order to be included in the meta-analysis, all the trial had to be RCTs and they should have compared the risk of SSI between using wound protectors and not using wound protector to cover the wound edges in colorectal resection.

### 2.3 Collection of the data

All the data from the included trials were examined by two independent reviewers and a predefined meta-analysis data form was used to extract the information. The data obtained from both reviewers was compared, resulting in accepted inter-reviewer agreement. The data included list of the authors, title of the published study, journal of publication, country and year of the publication, testing sample size (with sex differentiation if applicable), the number of patients in each group based on the use of wound protector, treatment protocol for each intervention and postoperative SSI.

### 2.4 Evidence map and synthesis

The statistical analysis was performed via software package RevMan 5.3 [15,16] provided by the Cochrane Collaboration. The summated outcome for binary data was defined by using the odds ratio (OR) with a 95% confidence interval while for continuous data variable the standardised mean difference with 95% CI was used. The random-effects model [17,18] was used to determine the combined outcomes results of both binary and continuous variables. Heterogeneity among included trials was analysed using the chi^2^ test, with significance set at p < 0.05, and was measured [19] using I^2^ test with a maximum value of 30% identifying low heterogeneity [19].The Mantel-Haenszel method was used for the calculation of OR under the random effect model [20] analysis. In a sensitivity analysis, 0.5 was added to each cell frequency for trials in which no event occurred in either the treatment or control group, according to the method recommended by Deeks et al. [21] If the standard deviation was unavailable, then it was calculated according to the guidelines provide by the Cochrane Collaboration [17] This method consisted of presuming that both groups had the same variance, which may not have been true, and variance was either estimated from the range or from the p-value. The estimate of the difference between both methods was pooled, depending upon the effect weights in results decided by each trial estimate variance. A forest plot was used for the graphical display of the results. The square around the estimate stood for the accuracy of the estimation (sample size), and the horizontal line represented the 95% CI. The methodological quality of the included trials was initially assessed using the published guidelines of Jaddad et al. and Chalmers et al. [22,23].

### 2.5 End point

Postoperative SSI following colorectal resection was analysed as the primary end point of this meta-analysis comparing the use of wound protector (WP) group versus non wound protector (NWP) group.

### 2.6 PRISMA 2020 statement compliance

The conduction of this systematic review, writing the manuscript and submission work is in compliance with the PRISMA criteria [24]. The AMSTAR 2 criteria to assess the quality of this systematic review was used and was more than 95% satisfactory [25].

## 3 Results

**Fig. 1:**
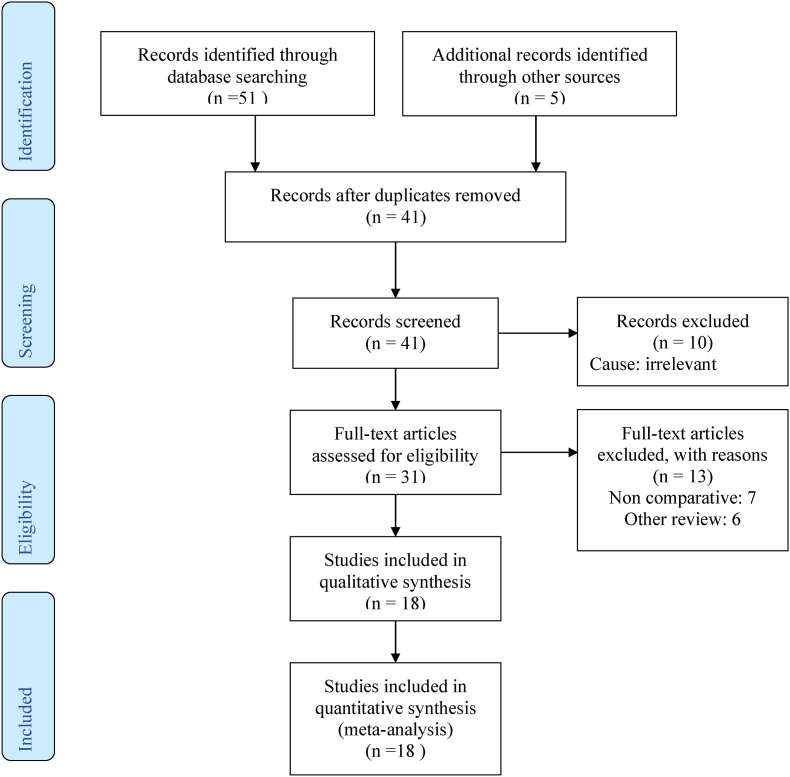
Prisma flow chart showing literature search outcomes.

Forty-one studies were found in the search of the standard medical database after removing duplicated ones. After a thorough examination 10 were excluded because they were found irrelevant. The remaining 31 studies were further assessed and only 18 trials were found to be suitable to be included in the meta-analysis (Fig. 1).

### 3.1 Features of included studies and patients

Twelve RCTs [[26], [27], [28], [29], [30], [31], [32], [33], [34], [35], [36], [37]] on 2425 patient were included in this meta-analysis after fulfilling the inclusion criteria. The PRIMA flow chart in trial search, trial deletion, trial selection and inclusion are given in Fig. 1. The included trials were conducted in Germany [26,27,32,33], Malaysia [28], UK [29,35], Japan [30,31], Sweden [34], Australia [36] and Mexico [37]. All trials were performed between 1984 [34] and 2020 [37]. The number of patients differs in each trial. The lowest number of patient was found to be 41 [37], while the highest number of patient was 594 [33]. Patients who participated in this meta-analysis were adults who underwent colorectal resection. All patients were informed about the trial before participation. Main characteristics of the included RCTs are given in Table 1 and the treatment protocol adopted in each of the trial is given in Table 2.

**Table 1:** Characteristic of included trials.

Study	Year	Country	Number of patients	Male to female ratio	Mean age
Baier [24]	2012	Germany	199	Not reported	103 96
Batz [25]	1987	Germany	50	Not reported	Not reported
Cheng [26]	2012	Malaysia	64	wound protector group: M:20 F:1 4 Non wound protector group: M: 13 F: 17	Wound protector group: 65 (22–83) Non wound protector group: 58.5 (39–86)
Gamble [27]	1984	England	56	Wound protector group: M: 11 F: 16 Non wound protector group: M: 16 F: 16	Wound protector group: 66 Non wound protector group: 65
Horiuchi [28]	2007	Japan	221	wound protector group: M: 61 F: 50 Non wound protector group: M:63 F:74	wound protector group: 67.0± 11.6 Non wound protector group: 64.6 ± 11.4
Kobayashi [29]	2019	Japan	102	Wound protector group: M: 29 F: 21 Non wound protector group: M: 29 F: 21	wound protector group: 69.5 Non wound protector group: 68.5
Lauscher [30]	2012	Germany	93	wound protector group: M:17 F: 29 Non wound protector group: M:17 F: 30	wound protector group: 50.1 ± 17.8 Non wound protector group: 48.5 ± 16.5
Mihaljevic [31]	2014	Germany	594	wound protector group: M: 168 F: 132 Non wound protector group: M: 169 F: 129	wound protector group: 69.0 (19–95) Non wound protector group: 67.0 (29–90)
Nystrom [32]	1984	Sweden	140	Not reported	wound protector group: 59 Non wound protector group: 60
Pinkney [33]	2013	UK	735	Wound protector group: M: 200 F:176 Non wound protector: M:193 F:180	Wound protector group: 66.4 (54.8–74.7) Non Wound protector group: 64.2 (55.5–72.8)
Reid [34]	2010	Australia	130	Wound protector group: M: 37 F: 27 Non wound Protector group: M: 41 F: 25	Wound protector group: 64.2 (14.8) Non wound Protector group: 63.1 (13.1)
Salgado-Nesme [35]	2020	Mexico	41	Wound protector group: M: 10 F: 11 Non wound Protector group: M: 5 F: 15	Wound protector group: 53.62 ± 22.98 Non wound Protector group: 57.5 ± 19.26

**Table 2:** Treatment adopted in each trial.

Study	Type of surgery	Intervention	Control
Baier [24]	laparotomy for any reason other than appendectomy and ostomy reduction	3MTM Steri-DrapeTM ring drape	wet cloth towels
Batz [25]	Colorectal Surgery	Single ring	Without ring drape With incision drape
Cheng [26]	elective colorectal resections via a standardized midline incision	ALEXIS O-Ring retractor	comprised four abdominal packs and Balfour retraction.
Gamble [27]	Elective colonic surgery	The plastic ring drape consists of flexible, semi-rigid plastic ring to the outer rim of which is welded a plastic sheet (single ring)	Drape was not used
Horiuchi [28]	Non-traumatic gastrointestinal surgery, laparoscopic surgery and minor surgery excluded open appendectomy	The Alexis retractor (dual ring)	Wound margin left untreated
Kobayashi [29]	elective open surgery for colorectal disease	Wound edge protector	no wound edge protector
Lauscher [30]	elective laparoscopic colorectal resection	plastic wound Ring drape	Without wound ring drapes
Mihaljevic [31]	elective open abdominal surgery requiring a median or transverse laparotomy	wound edge coverage	surgical towels
Nystrom [32]	Elective colorectal surgery involving opening the bowel	Op-drape (single ring)	Without Drape
Pinkney [33]	Laparotomy	Standard intraoperative care plus use of wound edge protector.	Standard intraoperative care
Reid [34]	Open colorectal surgery	This wound protector – Alexis (dual ring)	Wound retraction was achieved by retractors routinely used
Salgado-Nesme [35]	emergency open surgery	Alexis O ring	Without Alexis O Ring

**Table 3:** Qualities of included trials.

Study	Randomization Technique	Blinding	Concealment	Intention to treat
Baier [24]	Not reported	Non blinding	Not reported	Not reported
Batz [25]	Low	Not reported	Not reported	Not reported
Cheng [26]	Via sealed envelop	double-blind	Not reported	Not reported
Gamble [27]	Not reported	Not reported	Not reported	Not reported
Horiuchi [28]	Not reported	Assessor blind	Not reported	Not reported
Kobayashi [29]	Minimization randomization	Single blinded	Not reported	Not reported
Lauscher [30]	Via unstratified computer-generated randomization	Not reported	Not reported	Not reported
Mihaljevic [31]	computer- generated using the standard continuous uniform distribution	Double blinded	Via sealed envelops	reported
Nystrom [32]	Not reported	Not reported	Not reported	Not reported
Pinkney [33]	secure online system provided by the University of Birmingham	Double blinded	centralised secure web based system in a 1:1 ratio	Reported
Reid [34]	Via computer generated sequence allocation	Double blinded	opaque envelopes opened by a third party.	Reported
Salgado-Nesme [35]	1:1 randomization allocation ratio.	double-blind	Not reported	Not reported

### 3.2 Methodological characteristic of chosen studies

The Mantel-Haenszel random effects model was used to compute robustness and susceptibility to any outlier among these trials (see Table 3). The randomization technique was reported in 7 trials [28,[31], [32], [33],[35], [36], [37]]. Concealment was reported in 3 RCTs [33,35,36]. 5 of the included studies were double blinded [28,33,[35], [36], [37]] while the rest of the trials were either single blinded or the blinding is not reported. Statistically significant heterogeneity (clinical and methodological diversity) was seen among all these trials, but the random effects model analysis was used to counteract the issues related to this.

### 3.3 End point analysis outcome

**Fig. 2:**
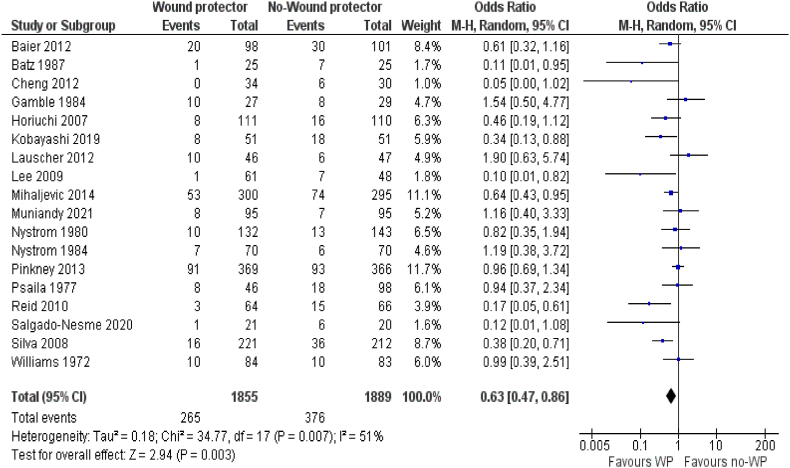
Forest plot showing the incidence of post operative surgical siter infection after colorectal resection. The outcome is presented as odd ratio with 95% confidence interval.

In the random effects model analysis, the use of WP during CRR was associated with the reduced risk of SSI [odds ratio 0.60, 95% CI (0.41–0.90), z = 2.49, P = 0.01]. However, there was significant heterogeneity (Tau^2^ = 0.22; Chi^2^ = 25.87, df = 11; (p = 0.007; I^2^ = 57%) among included studies (see Fig. 2).

## 4 Discussion

CRR are associated with high SSIs with a reported incidence of up to 20% [[38], [39], [40]], which is also considered the highest among elective operations [41]. Therefore, several precautions and measures were developed -pre, intra and post-operatively- over the past years to reduce the risk of post operative infections. Using single or double ringed circumferential wound protector in colorectal resection has been shown to be effective in reducing the SSIs. The results of this current meta-analysis of 12 RCTS on 2425 concurred with the results from previously published meta-analysis which showed the use of dual ring is effective in reducing SSI in lower gastrointestinal surgery [42]. This meta-analysis contains only RCTs and updated trials which were published in the last 5 years.

There are several limitations of this study. Firstly, it does not examine the difference in the rates of SSIs between different types of wound protectors. Moreover, it does not differentiate between laparoscopic and open colorectal resection. There was also significant heterogeneity among included trials which can generate bias. Therefore, a large multicentric RCTs is needed to compare the different types of wound protectors used in order to establish which wound protector is the best to be used to reduce the rate of infections. Comparison between open and laparoscopic colorectal resection would be helpful in determining the role of wound protector in relation to SSI in each group.

## Funding

None.

## Annals of medicine and surgery

The following information is required for submission. Please note that failure to respond to these questions/statements will mean your submission will be returned. If you have nothing to declare in any of these categories then this should be stated.

## Ethical approval

Not required.

## Please state any sources of funding for your research

None.

## Author contribution

Hussameldin M Nour: Idea conception, literature search, trial selection, data extraction, writing - original draft. Amiya Ahsan: literature search, trial selection, data extraction. Dimitra V. Peristeri: literature search, trial selection, data extraction, review & editing. Samuelson E Osifo: literature search, trial selection, data extraction. Mr Krishna K Singh: Writing - original draft, Writing - review & editing, Formal analysis. Muhammad S. Sajid: Data approval, data analysis, manuscript review and approval, supervision of the project.

## Registration of research studies

This research has been registered with a Research Registry UIN, registration unique ID: reviewregistry1447.

## Guarantor

Mr Muhammad S. Sajid.

## Consent

Not required.

## Provenance and peer review

Not commissioned, externally peer-reviewed.

## Declaration of competing interest

The authors declare that they have no known competing financial interests or personal relationships that could have appeared to influence the work reported in this paper.
